# Management of a Low-Energy Penetrating Brain Injury Caused by a Nail

**DOI:** 10.1155/2016/4371367

**Published:** 2016-06-26

**Authors:** V. R. Ferraz, G. B. Aguiar, J. L. Vitorino-Araujo, G. L. Badke, J. C. E. Veiga

**Affiliations:** ^1^Neurosurgery Residency Program, Discipline of Neurosurgery, Santa Casa de Sao Paulo School of Medical Sciences, Rua Doutor Cesário Mota Júnior 112, 01220-0202 Sao Paulo, SP, Brazil; ^2^Discipline of Neurosurgery, Santa Casa de Sao Paulo School of Medical Sciences, Rua Doutor Cesário Mota Júnior 112, 01220-0202 Sao Paulo, SP, Brazil

## Abstract

Low-energy penetrating nail injury to the brain is an extremely rare neurosurgical emergency. The most common cause of nail gun injury is work related accidents; other causes result from accidental firing of a nail gun, suicide attempts by firing nail guns into the brain, and bomb blasts containing pieces of nails. Neurosurgical treatment performed by craniotomy still seems to be the safest one; there are reports of complications such as subdural hematoma and intraparenchymal hemorrhages following the blind removal of foreign bodies leading to suggestions that all penetrating foreign bodies should be removed under direct vision. We report a rarely described neurosurgical approach for removal of a penetrating nail from the brain and skull without evidence of associated hematoma and other brain lesions.

## 1. Introduction

Low-energy penetrating nail injury to the brain is an extremely rare neurosurgical emergency. The most common cause of nail gun injury is work related accidents; other causes result from accidental firing of a nail gun, suicide attempts by firing nail guns into the brain, and bomb blasts containing pieces of nails. Except for the bomb blast, the prognosis of patients with nail injuries to the brain is good and they have a better outcome than the gunshot injuries by the fact that these lesions caused by nails are generated by low energetic kinetic mechanisms [1, 2].

## 2. Material and Methods

In this paper we report a rarely described neurosurgical approach for removal of a penetrating nail from the brain and skull without evidence of associated hematoma and other brain lesions. The patient and his family were informed about this paper and agreed to the publication of medical information about the patient.

## 3. Results: Case Report

Male patient, 55 years old, previously healthy, farm worker, was plowing a piece of land when the plowshare hits a nail on the grass which hit him in the right frontotemporal region. The patient had no neurological deficits on admission, four hours after the accident, lucid and oriented. On examination, he was not pale and not febrile. Basic investigations included full blood count which showed normal hemoglobin, normal platelet, and total white cells count. Creatinine, electrolytes, liver enzymes, and blood glucose levels were normal.

**Figure 1:**
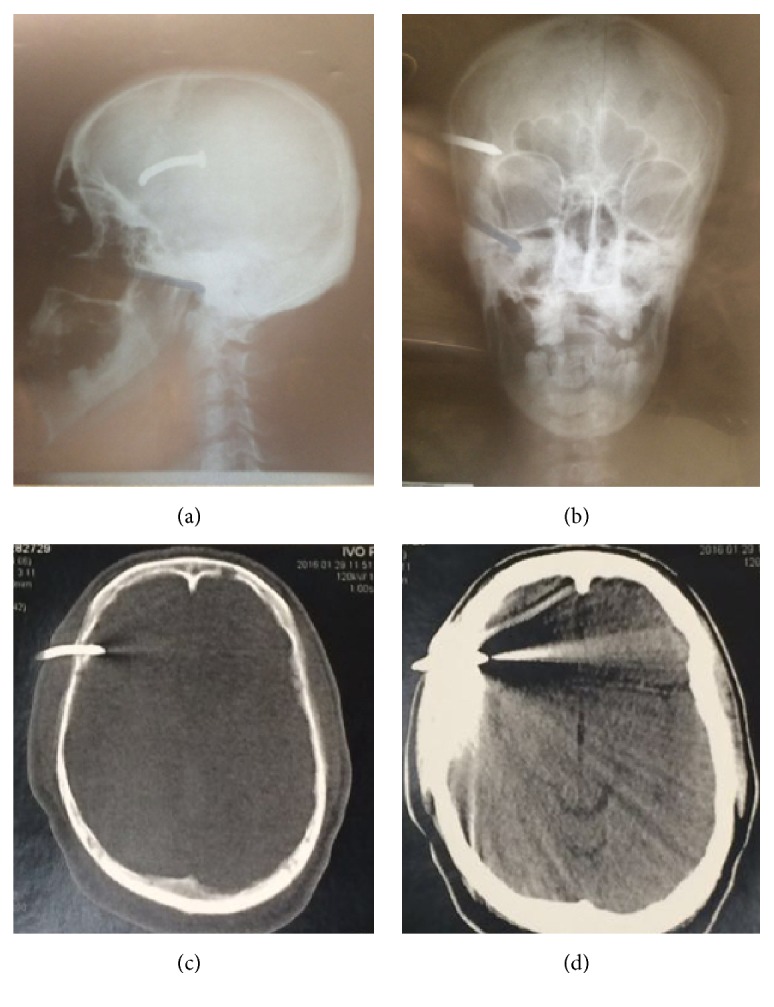
X-ray and head CT showing the presence of a nail on the frontotemporal region. (a) Preoperative simple sagittal skull X-ray showing a curvilinear, 7 cm long, oblique oriented nail in the skull. (b) Preoperative simple coronal skull X-ray showing a nail piercing the skull on the right frontotemporal region. (c) Axial head CT (bone window) showing one radiopaque nail which has penetrated both tables of skull and entered the brain parenchyma. (d) Axial head CT (soft tissue window) showing one radiopaque nail which has penetrated both tables of skull and entered the brain parenchyma.

**Figure 2:**
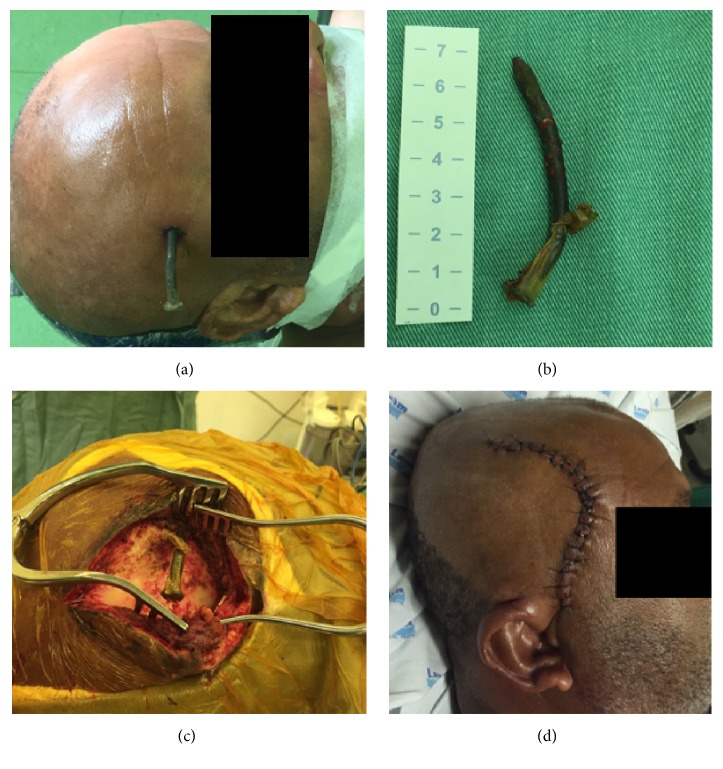
Intraoperative and postoperative figures related to the surgical procedure. (a) Patient underwent general anesthesia; local examination of the head after shaving revealed one curvilinear nail head on the right frontotemporal region with no active signs of bleeding. (b) Curvilinear 7 cm nail was removed. (c) Frontotemporal bone and nail exposure after the skin and subcutaneous incision. (d) Postoperative skin surgical scar with 13 cm length.

Local examination of the head after shaving revealed one nail head on the right frontotemporal region with no active signs of bleeding. The precise location of the nail was further delineated by the radiological study with the X-ray and computed tomography (CT) of head. Skull radiographs revealed a 7 cm, curvilinear, oblique oriented nail-shaped foreign body piercing the brain on the right frontotemporal region (Figures 1(a) and 1(b)). CT showed an object of metallic density that has penetrated the skull and entered the brain parenchyma on the frontotemporal region (Figures 1(c) and 1(d)). The plan was to treat the nail wound surgically. An informed consent was obtained and under general anesthesia a 13 cm curved incision was made across the nail head on its two sides. A small burr hole was created close to the nail and a small craniotomy was performed. The nail became loose and was held and pulled out very slowly and gently. The nail was about 7 cm long and after its removal, copious washing of the craniotomy area was performed with heated saline from a 20 mL syringe (Figures 2(a), 2(b), and 2(c)). The dural defect was repaired with nonabsorbable suture. There were no complications during surgery, and the patient was submitted to therapy with broad spectrum antibiotics and prophylaxis for seizures and tetanus. Postoperative skin surgical scar has remained good with no signs of infection (Figure 2(d)).

## 4. Discussion

Nail impalement injury to the brain is a rare neurosurgical emergency and the most common cause of is work related accident; other causes are infrequent and include criminal assaults, war injuries caused by explosions of bombs containing nails, and deliberate self-harm and suicide using nail guns. The kinetic energy of launched nail and the location of the nail in head are important determinant of the extent of nail gun related head injuries [3, 4]. In most cases, patients survive with a good neurological outcome, and the prognosis is better if the structural damage to the brain stem and major cerebral vessels is minimal [5].

Neuroimaging is vital in any penetrating head injury for surgical decision making, providing better planning of the type of surgery. All over the world the head CT scanning of the brain is now the primary modality used in the neuroradiologic evaluation of patients with penetrating head injury; this is because CT scanning is quick and provides the characterization of the missile trajectory, evaluation of the extent of brain injury, detection of the bone fragments in the brain even the smallest ones, and detection of intracranial hematomas and mass effects [6, 7].

The risk of local wound infections, meningitis, ventriculitis, or cerebral abscess is particularly high among penetrating head injury patients because of the presence of contaminated foreign objects, skin, hair, and bone fragments driven into the brain tissue along the projectile track. Despite many controversies regarding use of prophylactic antibiotics, broad spectrum antibiotics are appropriate for patients with penetrating brain injury [8, 9].

Neurosurgical treatment performed by craniotomy still seems to be the safest one; there are reports of complications such as subdural hematoma and intraparenchymal hemorrhages following the blind removal of foreign bodies leading to suggestions that all penetrating foreign bodies should be removed under direct vision [1, 4, 10]. Craniotomy must be performed around the nailing entry site enabling a safe removal of the nail together with the bone flap; all of the bone fragments should be removed from the brain with brain navigation if necessary and a lot of washing in the operating field for removal of tissue and skin debris for preventing future brain abscess.

## 5. Conclusion

Although low-energy penetrating brain injuries caused by nails are very rare, they can be managed properly if the patient is adequately assessed and treated by a medical team specialized in the treatment of traumatic brain injury and efficient care performed as soon as possible.
